# Individual behaviour self-regulation and emotional reactions among patients living with HIV during the second wave of the new coronavirus pandemic in Russia

**DOI:** 10.1192/j.eurpsy.2023.1723

**Published:** 2023-07-19

**Authors:** V. I. Rozhdestvenskiy, V. V. Titova, I. A. Gorkovaya, D. O. Ivanov, Y. S. Aleksandrovich

**Affiliations:** Department of Psychosomatics and Psychotherapy, Saint Petersburg State Pediatric Medical University, Saint Petersburg, Russian Federation

## Abstract

**Introduction:**

During the COVID-19 pandemic, the need to adapt to rapidly changing external conditions has increased dramatically. Predictors of successful adaptation can be the degree of development of individual self-regulation and its profile. The emotional state depends on successful adaptation.

**Objectives:**

The study aimed to examine the individual self-regulation of behaviour and emotional reactions among patients living with HIV in Russia.

**Methods:**

The data were collected from February to July 2021 using a Google form developed by us. Fifty-nine HIV-positive patients participated in the study. To diagnose the development of individual self-regulation and its profile, we used the Self-Regulation Style Questionnaire, to study depression, anxiety, and stress — DASS-21 adapted for use in Russia.

**Results:**

We found that 10 % of respondents had a low overall level of self-regulation, 53 % had an average level, and 37 % had a high level. The average individual profile was as follows: predominance of planning (M = 6.24±1.90) over modelling (M = 5.69±1.90), programming (M = 5.93±1.66), and evaluating results (M = 5.78±1.60), which were approximately at the same level. Flexibility (M = 6.58±1.90) and autonomy (M = 5.56±2.08) scores were in the average normal range. Only two correlations were found: modelling was negatively associated with depression (r_xy_ = -0.336, p < 0.01) and anxiety (r_xy_ = -0.275, p < 0.05).

**Conclusions:**

Awareness and adequacy of perceptions of changes in external and internal significant conditions contribute to a favourable emotional status among people living with HIV.

**Disclosure of Interest:**

None Declared

